# Reduced Incidence of Carbapenem-Resistant *Klebsiella pneumoniae* Infections in Cardiac Surgery Patients after Implementation of an Antimicrobial Stewardship Project

**DOI:** 10.3390/antibiotics8030132

**Published:** 2019-08-28

**Authors:** Daniele Roberto Giacobbe, Antonio Salsano, Filippo Del Puente, Francesco Campanini, Giovanni Mariscalco, Anna Marchese, Claudio Viscoli, Francesco Santini

**Affiliations:** 1 Dipartimento di Scienze della Salute (DISSAL), University of Genoa, 16132 Genoa, Italy; 2 Clinica Malattie Infettive, Ospedale Policlinico San Martino—IRCCS, 16132 Genoa, Italy; 3 Dipartimento di Scienze Chirurgiche e Diagnostiche Integrate (DISC), University of Genoa, 16132 Genoa, Italy; 4 Divisione di Cardiochirurgia, Ospedale Policlinico San Martino-IRCCS, 16132 Genoa, Italy; 5 Department of Cardiac Surgery, Glenfield Hospital, University Hospitals of Leicester, Leicester LE3 3QP, UK; 6 Unità di Microbiologia, Ospedale Policlinico San Martino—IRCCS, 16132 Genoa, Italy

**Keywords:** Klebsiella, stewardship, postoperative infections

## Abstract

Infections due to carbapenem-resistant *Klebsiella pneumoniae* (CR-Kp) are associated with increased mortality in cardiac surgery patients. In this short communication, we report on the changes in the incidence of CR-Kp colonization and CR-Kp infection in cardiac surgery patients from 2014 to 2018 in a teaching hospital in Italy, after the implementation of an antimicrobial stewardship project in 2014. During the study period, 2261 patients underwent open-heart surgery. Of them, 130 were found to be colonized by CR-Kp (5.7%) and 52 developed a postoperative CR-Kp infection (2.3%). The crude in-hospital mortality in patients with CR-Kp infections was 48% (25/52). The incidences of both CR-Kp colonization (incidence rate ratio (IRR) 0.82, 95% confidence intervals (CI) 0.78–0.86, *p* < 0.001) and CR-Kp infection (IRR 0.76, 95% CI 0.69–0.83, *p* < 0.001) considerably decreased over the study period. This encouraging result should prompt further concerted efforts, directed towards retaining the positive impact of stewardship and infection-control interventions on CR-Kp-related morbidity in the long term.

## 1. Introduction

Infections due to carbapenem-resistant *Klebsiella pneumoniae* (CR-Kp) are associated with increased mortality in cardiac surgery patients [1]. In most cases, development of CR-Kp infection follows CR-Kp intestinal colonization, although it may also follow colonization of other body sites [2,3].

To curtail mortality of infections due to these difficult-to-treat resistant bacteria, possible non-mutually exclusive interventions are: (i) Development of novel antimicrobial options for effectively treating CR-Kp infections [4]; (ii) implementation of infection-control measures for reducing in-hospital transmission of CR-Kp [5,6]; (iii) implementation of antimicrobial stewardship measures, aimed both at reducing mortality of CR-Kp infection (by improving the adequate use of antibiotics) and reducing the incidence of CR-Kp colonization and infection (by relieving selective pressure for further development and spread of resistance) [5,7].

In this short communication, we report on the changes in the incidence of CR-Kp colonization and CR-Kp infection in cardiac surgery patients after the implementation of an antimicrobial stewardship project in a teaching hospital in Italy.

## 2. Results

From 2014 to 2018, 2261 patients underwent open-heart surgery. Surgery encompassed 571 isolated coronary artery bypass graftings (CABG), 749 isolated valvular procedures, 250 replacements of thoracic aorta, and 691 other procedures, including combined procedures, pericardiectomy, and excisions of cardiac tumors. Isolated aortic valve replacement was performed through upper sternotomy, the other procedures by means of median sternotomy. Of 571 CABG, 9 were off-pump (1.5%).

During the study period, 130/2261 patients were found to be colonized by CR-Kp (5.7%) by means of systematic screening through rectal swabbing (100/130, 76.9%) or bronchoalveolar lavage (30/130, 23.1%). Colonization was detected preoperatively and postoperatively in 11.5% (15/130) and 88.5% (115/130) of cases, respectively (detailed timing of colonization is available in Supplementary Figure S1). Overall, 52/2261 patients developed a postoperative CR-Kp infection (2.3%). CR-Kp infection occurred at a median time of 18 days after surgery (interquartile range 7-34). The distribution of the different types of CR-Kp infections was as follows: 18 ventilator-associated pneumonia (VAP) (34.6%); 16 bloodstream infections (BSI) (30.8%); 9 sternal wound infections (17.3%); 5 VAP plus BSI (9.6%); 4 urinary tract infections (UTI) (7.7%). The majority of CR-Kp infections followed CR-Kp colonization (40/52, 76.9%). The crude in-hospital mortality in patients with CR-Kp infections was 48% (25/52). Stratification of types of infection in the different study years is available in Supplementary Table S1.

**Figure 1:**
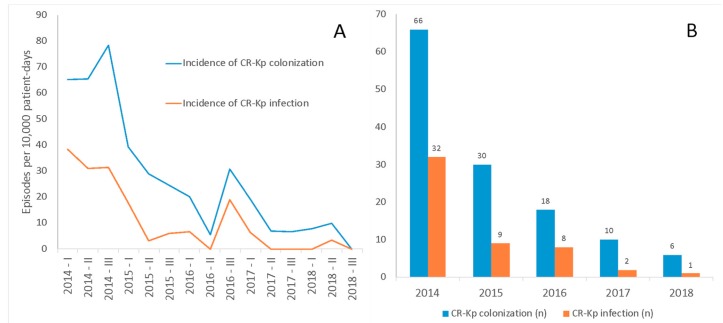
Trends in the incidence of colonization and infection by carbapenem-resistant Klebsiella pneumoniae (CR-Kp) in cardiac surgery patients over the study period. Panel **A**: Changes over quadrimesters in the incidence of CR-Kp colonization (incidence rate ratio (IRR) 0.82, 95% confidence intervals (CI) 0.78–0.86, *p* < 0.001) and CR-Kp infection (IRR 0.76, 95% CI 0.69–0.83, *p* < 0.001) were assessed through univariable, generalized linear models based on negative binomial regression (see study methods). Panel **B**: Number of episodes of CR-Kp colonization and CR-Kp infection during the study period. Overall, 40/130 colonized patients (30.8%) and 12/2131 non-colonized patients (0.6%) developed a postoperative CR-Kp infection.

The incidences of CR-Kp colonization and CR-Kp infection during the entire study period were 28 per 10,000 patient-days at risk and 11 per 10,000 patient-days at risk, respectively. The trends in the incidence of CR-Kp colonization and CR-Kp infection over the study period are shown in Figure 1. Incidences of both CR-Kp colonization (incidence rate ratio (IRR) 0.82, 95% confidence intervals (CI) 0.78–0.86, *p* < 0.001) and CR-Kp infection (IRR 0.76, 95% CI 0.69–0.83, *p* < 0.001) decreased over the study period.

Changes over the study period in the proportions/median values of other potential predictors of CR-Kp infection are displayed graphically in Supplementary Figure S2. A slight upward trend was observed for chronic obstructive pulmonary disease (COPD) (*p* = 0.005) and median cardiopulmonary bypass (CPB) time (*p* < 0.001), whereas a slight downward trend was observed for median age (*p* = 0.044).

## 3. Discussion

Infections due to CR-Kp are associated with high mortality (30–70%) [8]. One of the major reasons is the paucity of therapeutic options, since CR-Kp very frequently shows concomitant resistance to several other antibiotics, besides carbapenems [8]. Along with the development of novel effective agents, optimizing the use of already available antibiotics and reducing the intra-hospital dissemination of CR-Kp are paramount measures for counteracting the unfavorable impact of CR-Kp on patients’ health. After implementation of an antimicrobial stewardship project, consisting in the evaluation by an infectious diseases specialist of each prescription of antimicrobials impacting antibiotic resistance [5], we observed a significant decrease in the incidence of CR-Kp colonization and CR-Kp infection in cardiac surgery patients. Notably, there could also have been a favorable effect of the enhancement of CR-Kp-dedicated infection-control measures in our institution, including a possible long-term positive effect of the CR-Kp active surveillance protocol [5,9]. In this regard, it should nonetheless be noted that the introduction of CR-Kp dedicated infection-control measures (the use of patient-dedicated gowns and gloves in case of colonization/infection and active surveillance) predates the antimicrobial stewardship project (they were introduced in 2012), while the number of CR-Kp BSI remained constant until after the start of the antimicrobial stewardship project in 2014 (see limitations below for data on the other types of infections) [9]. Eventually, the relative contributions of infection-control measures and antimicrobial stewardship cannot be differentiated, but it is possible that both of them participated in determining the reduction of CR-Kp infections in an additive or synergistic way.

Considering the high crude in-hospital mortality in cardiac surgery patients with CR-Kp infections in the present study (48%), which is in line with reports in other surgical populations [3], our results are encouraging and testify to the beneficial effect of the concerted efforts of infectious diseases and infection-control specialists, cardiothoracic surgeons, pharmacists, and other professional figures in implementing and adhering to antimicrobial stewardship and infection-control initiatives aimed at reducing the spread of resistant organisms in our hospital.

It is also noteworthy that the distribution of other potential risk factors for CR-Kp infections in cardiac surgery patients [1] did not change over the study period, with a few exceptions: (i) A slight increase in the proportion of patients with COPD and in the median CPB time (which nonetheless should have theoretically favored an increase rather than a decrease in the incidence of CR-Kp infections) and (ii) a very slight trend towards reduced median age, that, although statistically significant, is unlikely to have substantially contributed to the observed incidence reduction.

There are some limitations in this study that could also reflect potential points of improvement of local antimicrobial stewardship initiatives. For example, we defined colonization as isolation of CR-Kp from rectal swab or respiratory samples, since they were collected systematically, but there were also a few other isolations of CR-Kp from urinary tract or surgical wounds in absence of signs and symptoms in infections. These types of colonization were not counted for defining colonization in this report, since samples were not collected systematically during the study period. However, their presence is helping us consider the possible implementation of the following additional measures: (i) Systematic screening through urine culture and surgical wound swabbing, for rapidly identifying these types of colonized patients, and in turn reducing their contribution in disseminate CR-Kp; (ii) a more frequent evaluation (and correction) of possible lacks of adherence to infection-control measures. In this regard, another limitation is the lack of information on the monitoring of hand hygiene and other infection-control measures, as well as the absence of molecular typing to provide more insights regarding the local CR-Kp epidemiology. Finally, an important limitation is the absence of data regarding CR-Kp colonization and infection in cardiac surgery patients in the period prior to the introduction of the antimicrobial stewardship project in 2014. However, stability of CR-Kp epidemiology in previous years is suggested by the constant annual rates of CR-Kp BSI in our hospital in the period 2011–2013, when CR-Kp was already endemic [9]. In conclusion, an important reduction in the incidence of CR-Kp colonization and infection was observed in cardiac surgery patients operated on in our institution from 2014 to 2018. However, this should not be seen as a definitive success, since reducing our attention might perilously renew the dissemination of these resistant bacteria. Rather, concerted efforts should always be directed towards further improving antimicrobial stewardship and infection-control interventions.

## 4. Materials and Methods 

This single-center, retrospective study was conducted at the San Martino Polyclinic Hospital-IRCCS, a 1200-bed teaching hospital in Genoa, Italy. Starting from May 2014, an antimicrobial stewardship program based on a combination of educational activities about the correct use of antimicrobials and standardized semi-restriction of prescriptions of antimicrobials deemed to have a major impact on antibiotic resistance (including also carbapenems) was implemented in the hospital [5].

CR-Kp colonization was defined as the first isolation of CR-Kp from a rectal swab or bronchoalveolar lavage during the hospital stay in patients with no signs or symptoms of infection. During the entire study period, routine active surveillance through rectal swabbing was performed at standard time points in all cardiac surgery patients, according to a local standard protocol: Before surgery; the first day after surgery; and every 7 days thereafter until discharge [1]. Routine bronchoalveolar lavage was systematically performed in all patients the first day after surgery. CR-Kp infection was defined according to the criteria of the Centers for Disease Control and Prevention (CDC) [10]. Carbapenem resistance was defined as the resistance to one or more carbapenems tested in our laboratory (i.e., ertapenem, imipenem, and meropenem). Identification of Kp and susceptibility testing were performed as previously described [1].

The primary objectives of the present study, conducted from 2014 to 2018, were to assess the trends in the incidence of CR-Kp colonization and CR-Kp infection starting from the year of implementation of the antimicrobial stewardship program (2014). Secondary objectives were to assess the trends in the proportions (for categorical variables) and in the median values (for continuous variables) of those factors which showed an independent association with the development of postoperative CR-Kp infection in a previous exploratory study we conducted in 2014 (i.e., age, female gender, increasing cardiopulmonary bypass time in minutes, chronic obstructive pulmonary disease [COPD], sequential organ failure assessment [SOFA] score immediate after surgery, preoperative mechanical ventilation, and prolonged postoperative mechanical ventilation) [1]. The complete definitions and statistical methods we employed in the present study are described in Supplementary methods.

## Supplementary Materials

The following are available online at https://www.mdpi.com/2079-6382/8/3/132/s1, Supplementary methods: definitions and statistical analysis; Supplementary Figure S1: Timing of carbapenem-resistant *Klebsiella pneumoniae* colonization; Supplementary Figure S2: Trends in the proportion or median value of other previously described, potential predictors of carbapenem-resistant *Klebsiella pneumoniae* infections in cardiac surgery patients; Supplementary Table S1: Observed types of postoperative carbapenem-resistant *Klebsiella pneumoniae* infections during the study period.

## References

[1] Salsano A., Giacobbe D.R., Sportelli E., Olivieri G.M., Brega C., Di Biase C., Coppo E., Marchese A., Del Bono V., Viscoli C. (2016). Risk factors for infections due to carbapenem-resistant *Klebsiella pneumoniae* after open heart surgery. Interact. Cardiovasc. Thorac. Surg..

[2] Giacobbe D.R., Del Bono V., Bruzzi P., Corcione S., Giannella M., Marchese A., Magnasco L., Maraolo A.E., Pagani N., Saffioti C. (2017). Previous bloodstream infections due to other pathogens as predictors of carbapenem-resistant *Klebsiella pneumoniae* bacteraemia in colonized patients: Results from a retrospective multicentre study. Eur. J. Clin. Microbiol. Infect. Dis..

[3] Giannella M., Bartoletti M., Morelli M.C., Tedeschi S., Cristini F., Tumietto F., Pasqualini E., Danese I., Campoli C., Lauria N.D. (2015). Risk factors for infection with carbapenem-resistant *Klebsiella pneumoniae* after liver transplantation: The importance of pre- and posttransplant colonization. Am. J. Transplant..

[4] Bassetti M., Peghin M., Vena A., Giacobbe D.R. (2019). Treatment of Infections Due to MDR Gram-Negative Bacteria. Front. Med..

[5] Giacobbe D.R., Del Bono V., Mikulska M., Gustinetti G., Marchese A., Mina F., Signori A., Orsi A., Rudello F., Alicino C. (2017). Impact of a mixed educational and semi-restrictive antimicrobial stewardship project in a large teaching hospital in Northern Italy. Infection.

[6] Li M., Wang X., Wang J., Tan R., Sun J., Li L., Huang J., Wu J., Gu Q., Zhao Y. (2019). Infection-prevention and control interventions to reduce colonisation and infection of intensive care unit-acquired carbapenem-resistant *Klebsiella pneumoniae*: A 4-year quasi-experimental before-and-after study. Antimicrob. Resist. Infect. Control.

[7] Rao V.P., Wu J., Gillott R., Baig M.W., Kaul P., Sandoe J.A.T. (2019). Impact of the duration of antibiotic therapy on relapse and survival following surgery for active infective endocarditis. Eur. J. Cardiothorac. Surg..

[8] Tumbarello M., Trecarichi E.M., De Rosa F.G., Giannella M., Giacobbe D.R., Bassetti M., Losito A.R., Bartoletti M., Del Bono V., Corcione S. (2015). Infections caused by KPC-producing *Klebsiella pneumoniae*: Differences in therapy and mortality in a multicentre study. J. Antimicrob. Chemother..

[9] Alicino C., Giacobbe D.R., Orsi A., Tassinari F., Trucchi C., Sarteschi G., Copello F., Del Bono V., Viscoli C., Icardi G. (2015). Trends in the annual incidence of carbapenem-resistant *Klebsiella pneumoniae* bloodstream infections: A 8-year retrospective study in a large teaching hospital in northern Italy. BMC Infect. Dis..

[10] CDC/NHSH Surveillance Definitions for Specific Types of Infections. https://www.cdc.gov/nhsn/pdfs/pscmanual/17pscnosinfdef_current.pdf.

