# Supplementary material for: Reduced Incidence of Carbapenem-Resistant Klebsiella pneumoniae Infections in Cardiac Surgery Patients after Implementation of an Antimicrobial Stewardship Project
Source: Antibiotics (Basel). 2019 Aug 28;8(3):132. doi: 10.3390/antibiotics8030132 (PMC6783823; doi:10.3390/antibiotics8030132)
Supplement: Supplementary file 1 [file antibiotics-08-00132-s001.zip › Supplementary methods.pdf]

Supplementary methods

Definitions

CR-Kp colonization was defined as the first isolation of at CR-Kp from rectal swab or bronchoalveolar lavage during hospital stay, in patients with no signs and symptoms of infection. During the entire study period, routine surveillance rectal swabbing was performed at standard time points in all cardiac surgery patients, according to a pre-defined ward protocol: before surgery; the first day after surgery; and every 7 days thereafter until discharge [1]. Routine bronchoalveolar lavage was systematically performed in all patients the first day after surgery. CR-Kp infection was defined according to the criteria of the Centers for Disease Control and Prevention (CDC) [2]. Carbapenem resistance was defined as resistance to one or more carbapenems tested in our laboratory (i.e. ertapenem, imipenem, and meropenem). Identification of Kp and susceptibility testing were performed as previously described [1]. Methods to detect CR-Kp gut colonization remained constant during the study period: chromogenic plates combined with bla_{KPC} gene detection.

The incidence of CR-Kp colonization was defined as the number of new colonization per 10,000 patient-days at risk of colonization (i.e., the cumulative number of patient-days without CR-Kp colonization). The incidence of CR-Kp infection was defined as the number of new colonization per 10,000 patient-days at risk of infection (i.e., the cumulative number of patient-days without CR-Kp infection).

Chronic obstructive pulmonary disease (COPD) was defined as long-term use of bronchodilators or steroids for lung disease. Prolonged mechanical ventilation was defined as postoperative mechanical ventilation >48 h.

Changes both in the incidence of CR-Kp colonization and CR-Kp infections and in the proportion and median values of other factors possibly influencing the development of CR-Kp infection over the study period were evaluated using 4-month periods as the unit of time.

Crude in-hospital mortality was measured in patients developing CR-Kp infections and defined as all-cause death occurring during hospital stay.

Statistical analysis

Changes in the incidence of CR-Kp colonization and CR-Kp infection over the study period were assessed through univariable, generalized linear models based on negative binomial regression, employing the glm.nb function in the lme4 package for R Statistical Software. In the CR-Kp colonization model, colonization events were the dependent variable, and patient-days at risk of colonization were included as an offset. In the CR-Kp infection model, infection events were the dependent variable, and patient-days at risk of infection were included as an offset. In both models, time in 4-month periods was the independent variable.

Changes over the study quadrimesters in the proportion of other potential, categorical predictors of CR-Kp infections in cardiac surgery patients were assessed by means of the Cochrane-Armitage test for trend in proportions, using the DescTools package for R Statistical Software. Changes over the study quadrimesters in the median values of continuous predictors of CR-Kp infections in cardiac surgery patients were assessed using the two-sided Jonckheere-Terpstra rank-based trend test included in the clinfun package for R Statistical Software.

References

[1] A. Salsano, D. R. Giacobbe, E. Sportelli, G. M. Olivieri, C. Brega, C. Di Biase et al. Risk factors for infections due to carbapenem-resistant klebsiella pneumoniae after open heart surgery. Interact Cardiovasc Thorac Surg 2016;23:762-68.

[2] Cdc/nhsn surveillance definitions for specific types of infections [cited 2019 Jun 10]. Available from: https://www.cdc.gov/nhsn/pdfs/pscmanual/17pscnosinfdef_current.pdf.
