# Supplementary material for: Reduced Incidence of Carbapenem-Resistant Klebsiella pneumoniae Infections in Cardiac Surgery Patients after Implementation of an Antimicrobial Stewardship Project
Source: Antibiotics (Basel). 2019 Aug 28;8(3):132. doi: 10.3390/antibiotics8030132 (PMC6783823; doi:10.3390/antibiotics8030132)
Supplement: Supplementary file 1 [file antibiotics-08-00132-s001.zip › Supplementary table S1.pdf]

Supplementary table S1. Observed types of postoperative carbapenem-resistant *Klebsiella pneumoniae* infections during the study period.

Year	Bloodstream infections		Isolated pneumonia	Urinary tract infection	Surgical wound infections	Total per year
	Isolated	With concomitant pneumonia				
**2014**	7	4	14	3	4	32
**2015**	3	0	2	0	4	9
**2016**	5	0	2	1	0	8
**2017**	0	1	0	0	1	2
**2018**	1	0	0	0	0	1
**Entire period**	16	5	18	4	9	52
