# Supplementary material for: Reduced Incidence of Carbapenem-Resistant Klebsiella pneumoniae Infections in Cardiac Surgery Patients after Implementation of an Antimicrobial Stewardship Project
Source: Antibiotics (Basel). 2019 Aug 28;8(3):132. doi: 10.3390/antibiotics8030132 (PMC6783823; doi:10.3390/antibiotics8030132)
Supplement: Supplementary file 1 [file antibiotics-08-00132-s001.zip › Supplementary figure S2.pdf]

Supplementary figure S2. Trends in the proportion or median value of other previously described, potential predictors of carbapenem-resistant *Klebsiella pneumoniae* infections in cardiac surgery patients

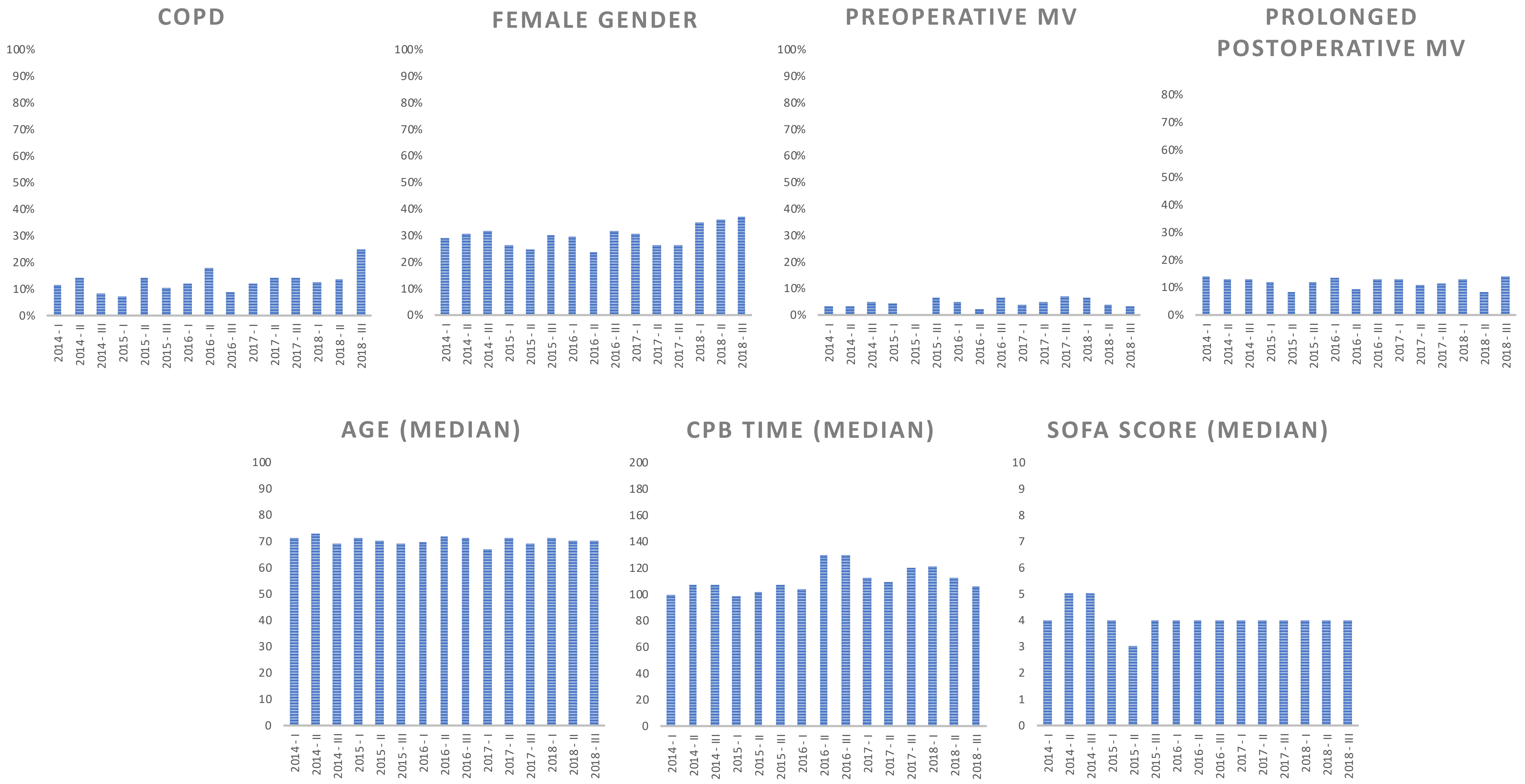

Figure S2 legend. Overall, 2261 patients underwent open-heart surgery during the study period. Trends over 4-month periods are reported as proportions (percentages) unless otherwise indicated. COPD, chronic obstructive pulmonary disease; MV, mechanical ventilation, SOFA, sequential organ failure assessment. CPB, cardiopulmonary bypass. Statistically significant upward trends were observed for COPD ($p = 0.005$) and CPB time ($p < 0.001$), whereas a statistically significant downward trend was observed for patients' age ($p = 0.044$). No other statistically significant trends were observed (female gender, $p = 0.115$; preoperative mechanical ventilation, $p = 0.266$; prolonged postoperative mechanical ventilation, $p = 0.728$); SOFA score, $p = 0.339$).
