# Supplementary material for: Reduced Incidence of Carbapenem-Resistant Klebsiella pneumoniae Infections in Cardiac Surgery Patients after Implementation of an Antimicrobial Stewardship Project
Source: Antibiotics (Basel). 2019 Aug 28;8(3):132. doi: 10.3390/antibiotics8030132 (PMC6783823; doi:10.3390/antibiotics8030132)
Supplement: Supplementary file 1 [file antibiotics-08-00132-s001.zip › Supplementary figure S1.pdf]

Supplementary figure S1. Timing of carbapenem-resistant *Klebsiella pneumoniae* colonization

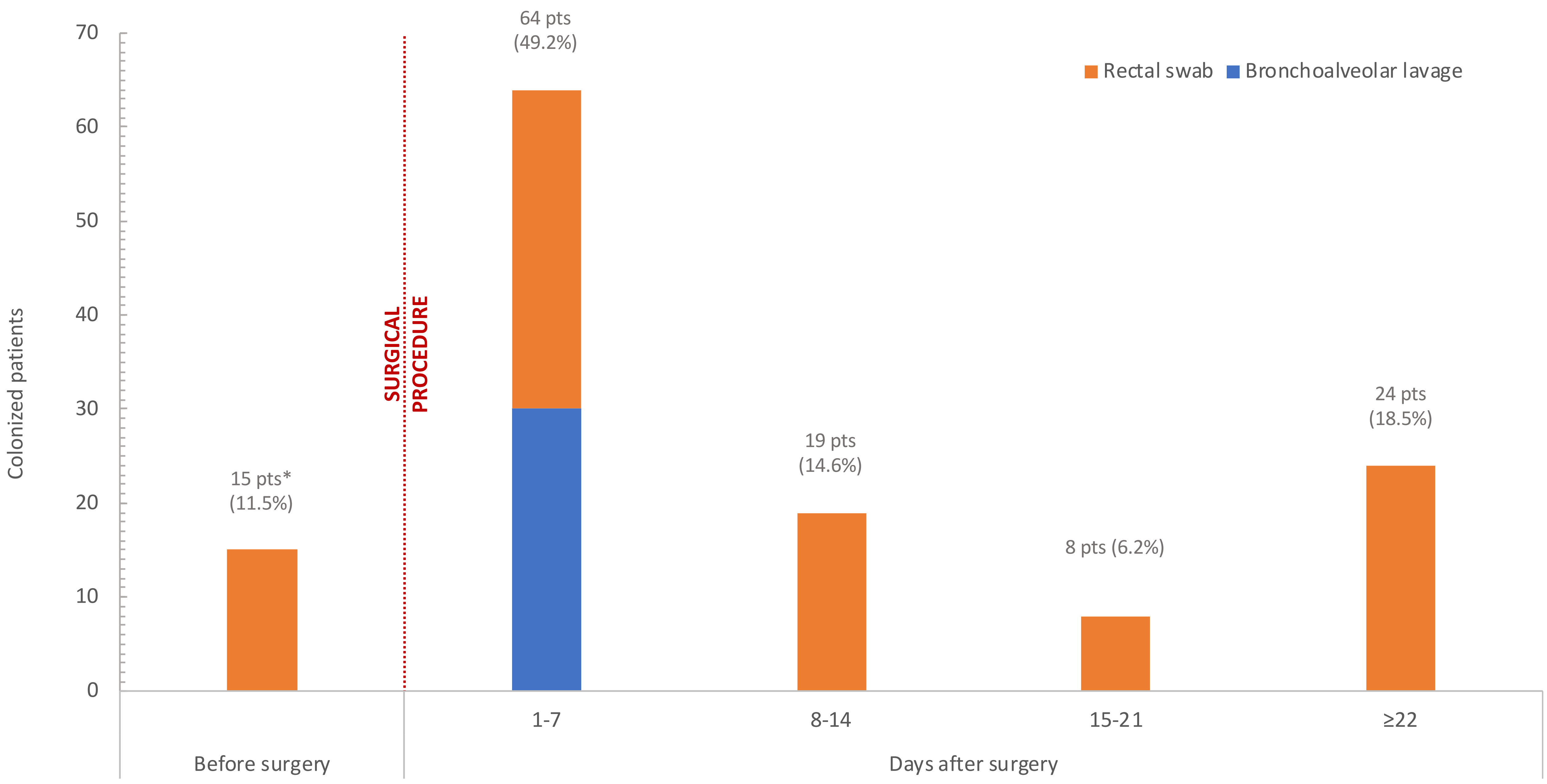

* in 7 cases colonization was known at hospital admission
